# (*E*)-(2-Chloro­benzyl­idene)amino 2-amino-4-chloro­benzoate

**DOI:** 10.1107/S1600536812003844

**Published:** 2012-02-17

**Authors:** Weiyan Yin, Zhi Wang, Ying Liang, Zi-Wen Yang

**Affiliations:** a Hubei Biopesticide Engineering Research Center, Hubei Academy of Agricultural Science, Wuhan 430064, People’s Republic of China

## Abstract

In the title compound, C_14_H_10_Cl_2_N_2_O_2_, the configuration about the C=N double bond is *E* and the dihedral angle between the benzene rings is 1.75 (5)°. An intra­molecular N—H⋯O inter­action generates an *S*(6) ring. In the crystal, mol­ecules are linked by N—H⋯O hydrogen bonds, resulting in [101] chains.

## Related literature

For background to 2-amino-4-chloro­benzoic acid derivatives, see: Jahnke *et al.* (2010 ▶); Lee *et al.* (2005 ▶). For a related structure, see: Seong *et al.* (2008 ▶).
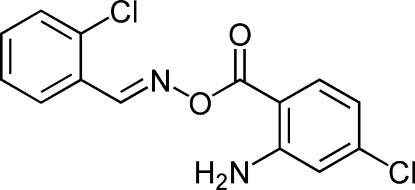


## Experimental

### 

#### Crystal data

C_14_H_10_Cl_2_N_2_O_2_
*M* *_r_* = 309.14
Monoclinic, 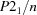

*a* = 7.4034 (5) Å
*b* = 23.8190 (15) Å
*c* = 7.6362 (5) Å
β = 96.382 (1)°
*V* = 1338.23 (15) Å^3^
*Z* = 4
Mo *K*α radiation
μ = 0.49 mm^−1^
*T* = 100 K
0.16 × 0.15 × 0.10 mm

#### Data collection

Bruker SMART APEX CCD diffractometer
Absorption correction: multi-scan (*SADABS*; Bruker, 2000 ▶) *T*_min_ = 0.926, *T*_max_ = 0.953
12076 measured reflections
3879 independent reflections
3637 reflections with *I* > 2σ(*I*)
*R*_int_ = 0.015

#### Refinement

*R*[*F* ^2^ > 2σ(*F* ^2^)] = 0.028
*wR*(*F* ^2^) = 0.084
*S* = 1.05
3879 reflections
182 parameters
H-atom parameters constrained
Δρ_max_ = 0.53 e Å^−3^
Δρ_min_ = −0.53 e Å^−3^

### 

Data collection: *SMART* (Bruker, 2000 ▶); cell refinement: *SAINT* (Bruker, 2000 ▶); data reduction: *SAINT*; program(s) used to solve structure: *SHELXS97* (Sheldrick, 2008 ▶); program(s) used to refine structure: *SHELXL97* (Sheldrick, 2008 ▶); molecular graphics: *SHELXTL* (Sheldrick, 2008 ▶); software used to prepare material for publication: *SHELXTL*.

## Supplementary Material

Crystal structure: contains datablock(s) global, I. DOI: 10.1107/S1600536812003844/hb6586sup1.cif

Structure factors: contains datablock(s) I. DOI: 10.1107/S1600536812003844/hb6586Isup2.hkl

Supplementary material file. DOI: 10.1107/S1600536812003844/hb6586Isup3.cml

Additional supplementary materials:  crystallographic information; 3D view; checkCIF report

## Figures and Tables

**Table 1:** Hydrogen-bond geometry (Å, °)

*D*—H⋯*A*	*D*—H	H⋯*A*	*D*⋯*A*	*D*—H⋯*A*
N2—H2*B*⋯O2	0.88	2.02	2.6653 (12)	130
N2—H2*A*⋯O2^i^	0.88	2.19	2.9332 (12)	142

Symmetry code: (i) 
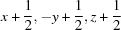
.

**Fig. 1.**
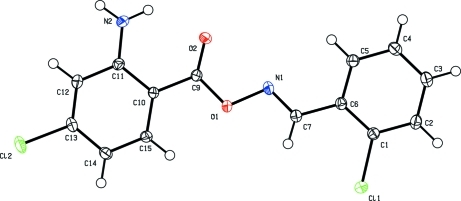
The molecular structure of the title compound, showing 30% probability displacement ellipsoids.

**Fig. 2.**
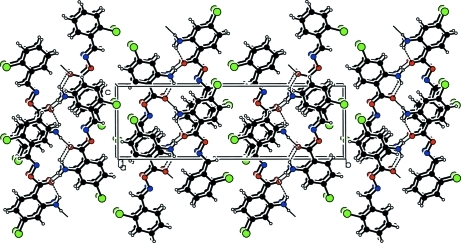
Crystal Packing diagram of (I). Hydrogen bonds are shown as dashed lines.

## supplementary crystallographic information

### Comment

2-Amino-4-chlorobenzoic acid derivatives show diverse biological properties such as inhibitor of the prostaglandin H2 synthase peroxidase activity (Lee *et al.*, 2005) and allosteric inhibitors of Bcr-Abl (Jahnke *et al.*, 2010). As a part of our studies of 2-aminobenzoic acid-containing compounds with potential biological activities, we report here the crystal structure of the title compound, (I) (Fig. 1).

The conformation of the N—H and the C=O bonds in the 2-aminobenzoic acid segment is similar to that observed in other 2-aminobenzoic acid compound (Seong *et al.*, 2008). The dihedral angles between the two phenyl rings is 1.75 (5)°. The molecular structure is linked by N—H···O hydrogen-bonds (Table 1).

### Experimental

Dicyclohexylcarbodiimide (1.1 g, 5.0 mmol) and 4-dimethylamiopryidine(0.25 g, 1.0 mmol) was added to a mixture of 2-chlorobenzaldehyde oxime (0.78 g, 5.0 mmol) and 2-amino-4-chlorobenzoic acid (0.86 g, 5.0 mmol) in dichloromethane (30 ml). The reaction mixture was stirred for 14 h at 353 k. The product was collected by filtration give a gray solid and recrystallization from its ether solution yielded colourless prisms of (I) after a few days.

### Refinement

The H atoms were placed in calculated positions (C—H = 0.93–0.97Å and N—H = 0.86 Å), and refined as riding with *U*_iso_ (H) = 1.2*U*_eq_(C, N).

### Crystal data

**Table:**

C_14_H_10_Cl_2_N_2_O_2_	*F*(000) = 632
*M**_r_* = 309.14	*D*_x_ = 1.534 Mg m^−^^3^
Monoclinic, *P*2_1_/*n*	Mo *K*α radiation, λ = 0.71073 Å
*a* = 7.4034 (5) Å	Cell parameters from 9848 reflections
*b* = 23.8190 (15) Å	θ = 2.8–32.0°
*c* = 7.6362 (5) Å	µ = 0.49 mm^−^^1^
β = 96.382 (1)°	*T* = 100 K
*V* = 1338.23 (15) Å^3^	Block, colorless
*Z* = 4	0.16 × 0.15 × 0.10 mm

### Data collection

**Table:**

Bruker SMART APEX CCD diffractometer	3879 independent reflections
Radiation source: fine-focus sealed tube	3637 reflections with *I* > 2σ(*I*)
Graphite monochromator	*R*_int_ = 0.015
φ and ω scans	θ_max_ = 30.0°, θ_min_ = 1.7°
Absorption correction: multi-scan (*SADABS*; Bruker, 2000)	*h* = −10→10
*T*_min_ = 0.926, *T*_max_ = 0.953	*k* = −33→32
12076 measured reflections	*l* = −10→10

### Refinement

**Table:**

Refinement on *F*^2^	Secondary atom site location: difference Fourier map
Least-squares matrix: full	Hydrogen site location: inferred from neighbouring sites
*R*[*F*^2^ > 2σ(*F*^2^)] = 0.028	H-atom parameters constrained
*wR*(*F*^2^) = 0.084	*w* = 1/[σ^2^(*F*_o_^2^) + (0.0495*P*)^2^ + 0.5103*P*] where *P* = (*F*_o_^2^ + 2*F*_c_^2^)/3
*S* = 1.05	(Δ/σ)_max_ = 0.001
3879 reflections	Δρ_max_ = 0.53 e Å^−^^3^
182 parameters	Δρ_min_ = −0.53 e Å^−^^3^
0 restraints	Extinction correction: *SHELXL97* (Sheldrick, 2008), Fc^*^=kFc[1+0.001xFc^2^λ^3^/sin(2θ)]^-1/4^
Primary atom site location: structure-invariant direct methods	Extinction coefficient: 0.0048 (10)

### Special details

**Table:**

Geometry. All e.s.d.'s (except the e.s.d. in the dihedral angle between two l.s. planes) are estimated using the full covariance matrix. The cell e.s.d.'s are taken into account individually in the estimation of e.s.d.'s in distances, angles and torsion angles; correlations between e.s.d.'s in cell parameters are only used when they are defined by crystal symmetry. An approximate (isotropic) treatment of cell e.s.d.'s is used for estimating e.s.d.'s involving l.s. planes.
Refinement. Refinement of *F*^2^ against ALL reflections. The weighted *R*-factor *wR* and goodness of fit *S* are based on *F*^2^, conventional *R*-factors *R* are based on *F*, with *F* set to zero for negative *F*^2^. The threshold expression of *F*^2^ > σ(*F*^2^) is used only for calculating *R*-factors(gt) *etc*. and is not relevant to the choice of reflections for refinement. *R*-factors based on *F*^2^ are statistically about twice as large as those based on *F*, and *R*- factors based on ALL data will be even larger.

### Fractional atomic coordinates and isotropic or equivalent isotropic displacement parameters (Å^2^)

**Table:**

	*x*	*y*	*z*	*U*_iso_*/*U*_eq_	
C1	0.45478 (14)	0.08217 (4)	0.17648 (13)	0.01523 (18)	
C2	0.36816 (14)	0.09752 (5)	0.01219 (13)	0.01805 (19)	
H2	0.3133	0.0698	−0.0659	0.022*	
C3	0.36279 (15)	0.15379 (5)	−0.03640 (14)	0.0197 (2)	
H3	0.3043	0.1646	−0.1483	0.024*	
C4	0.44295 (15)	0.19433 (5)	0.07849 (14)	0.0190 (2)	
H4	0.4383	0.2328	0.0453	0.023*	
C5	0.52956 (14)	0.17850 (4)	0.24135 (13)	0.01681 (19)	
H5	0.5854	0.2063	0.3184	0.020*	
C6	0.53611 (13)	0.12204 (4)	0.29440 (13)	0.01445 (18)	
C7	0.62742 (13)	0.10574 (4)	0.46828 (13)	0.01528 (18)	
H7	0.6660	0.0682	0.4918	0.018*	
C9	0.80555 (13)	0.16068 (4)	0.86039 (13)	0.01431 (18)	
C10	0.89159 (13)	0.13801 (4)	1.02756 (13)	0.01340 (17)	
C11	0.97488 (13)	0.17569 (4)	1.15683 (13)	0.01466 (18)	
C12	1.05960 (14)	0.15271 (4)	1.31596 (13)	0.01651 (19)	
H12	1.1176	0.1767	1.4044	0.020*	
C13	1.05789 (14)	0.09568 (4)	1.34250 (13)	0.01679 (19)	
C14	0.97615 (14)	0.05787 (4)	1.21752 (14)	0.01768 (19)	
H14	0.9772	0.0186	1.2394	0.021*	
C15	0.89387 (13)	0.07991 (4)	1.06104 (13)	0.01523 (18)	
H15	0.8374	0.0552	0.9739	0.018*	
Cl1	0.45712 (4)	0.011502 (10)	0.23325 (3)	0.01985 (8)	
Cl2	1.16302 (4)	0.069412 (12)	1.54105 (3)	0.02424 (8)	
N1	0.65290 (13)	0.14358 (4)	0.58580 (12)	0.01819 (18)	
N2	0.97461 (13)	0.23234 (4)	1.13648 (13)	0.02025 (19)	
H2A	1.0264	0.2539	1.2213	0.024*	
H2B	0.9226	0.2475	1.0385	0.024*	
O1	0.74622 (10)	0.11954 (3)	0.74289 (10)	0.01663 (15)	
O2	0.78875 (12)	0.21027 (3)	0.82487 (11)	0.02169 (17)	

### Atomic displacement parameters (Å^2^)

**Table:**

	*U*^11^	*U*^22^	*U*^33^	*U*^12^	*U*^13^	*U*^23^
C1	0.0162 (4)	0.0151 (4)	0.0144 (4)	0.0010 (3)	0.0017 (3)	−0.0007 (3)
C2	0.0188 (4)	0.0214 (5)	0.0136 (4)	0.0015 (4)	−0.0002 (3)	−0.0026 (3)
C3	0.0209 (5)	0.0236 (5)	0.0145 (4)	0.0046 (4)	0.0011 (4)	0.0016 (4)
C4	0.0220 (5)	0.0179 (4)	0.0173 (4)	0.0025 (4)	0.0034 (4)	0.0030 (4)
C5	0.0192 (4)	0.0158 (4)	0.0156 (4)	−0.0005 (3)	0.0027 (3)	−0.0001 (3)
C6	0.0147 (4)	0.0159 (4)	0.0127 (4)	0.0010 (3)	0.0018 (3)	0.0001 (3)
C7	0.0158 (4)	0.0157 (4)	0.0140 (4)	−0.0002 (3)	0.0002 (3)	0.0006 (3)
C9	0.0131 (4)	0.0141 (4)	0.0151 (4)	−0.0011 (3)	−0.0016 (3)	0.0003 (3)
C10	0.0129 (4)	0.0133 (4)	0.0134 (4)	−0.0004 (3)	−0.0014 (3)	0.0004 (3)
C11	0.0134 (4)	0.0142 (4)	0.0160 (4)	−0.0009 (3)	−0.0001 (3)	−0.0010 (3)
C12	0.0155 (4)	0.0195 (5)	0.0139 (4)	−0.0010 (3)	−0.0008 (3)	−0.0010 (3)
C13	0.0150 (4)	0.0217 (5)	0.0133 (4)	0.0003 (3)	−0.0002 (3)	0.0041 (3)
C14	0.0181 (4)	0.0157 (4)	0.0187 (5)	−0.0008 (3)	−0.0003 (4)	0.0040 (3)
C15	0.0152 (4)	0.0134 (4)	0.0166 (4)	−0.0012 (3)	−0.0005 (3)	0.0009 (3)
Cl1	0.02618 (14)	0.01429 (12)	0.01833 (13)	−0.00055 (8)	−0.00089 (9)	−0.00137 (8)
Cl2	0.02529 (14)	0.03026 (15)	0.01581 (13)	−0.00042 (10)	−0.00378 (10)	0.00817 (9)
N1	0.0215 (4)	0.0180 (4)	0.0137 (4)	0.0028 (3)	−0.0036 (3)	0.0012 (3)
N2	0.0244 (4)	0.0134 (4)	0.0210 (4)	−0.0021 (3)	−0.0062 (3)	−0.0016 (3)
O1	0.0210 (4)	0.0146 (3)	0.0130 (3)	0.0009 (3)	−0.0041 (3)	−0.0002 (2)
O2	0.0264 (4)	0.0138 (3)	0.0223 (4)	−0.0026 (3)	−0.0090 (3)	0.0035 (3)

### Geometric parameters (Å, º)

**Table:**

C1—C2	1.3928 (14)	C9—C10	1.4649 (13)
C1—C6	1.3980 (14)	C10—C15	1.4071 (13)
C1—Cl1	1.7378 (10)	C10—C11	1.4230 (13)
C2—C3	1.3902 (15)	C11—N2	1.3581 (13)
C2—H2	0.9500	C11—C12	1.4143 (14)
C3—C4	1.3923 (15)	C12—C13	1.3736 (14)
C3—H3	0.9500	C12—H12	0.9500
C4—C5	1.3866 (14)	C13—C14	1.3999 (15)
C4—H4	0.9500	C13—Cl2	1.7417 (10)
C5—C6	1.4039 (14)	C14—C15	1.3829 (14)
C5—H5	0.9500	C14—H14	0.9500
C6—C7	1.4735 (14)	C15—H15	0.9500
C7—N1	1.2712 (13)	N1—O1	1.4352 (11)
C7—H7	0.9500	N2—H2A	0.8800
C9—O2	1.2151 (12)	N2—H2B	0.8800
C9—O1	1.3677 (12)		
			
C2—C1—C6	121.62 (9)	C15—C10—C11	119.92 (9)
C2—C1—Cl1	118.01 (8)	C15—C10—C9	121.07 (9)
C6—C1—Cl1	120.37 (8)	C11—C10—C9	119.01 (9)
C3—C2—C1	119.34 (10)	N2—C11—C12	118.53 (9)
C3—C2—H2	120.3	N2—C11—C10	123.52 (9)
C1—C2—H2	120.3	C12—C11—C10	117.94 (9)
C2—C3—C4	120.20 (10)	C13—C12—C11	119.94 (9)
C2—C3—H3	119.9	C13—C12—H12	120.0
C4—C3—H3	119.9	C11—C12—H12	120.0
C5—C4—C3	119.92 (10)	C12—C13—C14	123.09 (9)
C5—C4—H4	120.0	C12—C13—Cl2	118.24 (8)
C3—C4—H4	120.0	C14—C13—Cl2	118.67 (8)
C4—C5—C6	121.13 (10)	C15—C14—C13	117.41 (9)
C4—C5—H5	119.4	C15—C14—H14	121.3
C6—C5—H5	119.4	C13—C14—H14	121.3
C1—C6—C5	117.79 (9)	C14—C15—C10	121.70 (9)
C1—C6—C7	121.54 (9)	C14—C15—H15	119.1
C5—C6—C7	120.67 (9)	C10—C15—H15	119.1
N1—C7—C6	117.81 (9)	C7—N1—O1	109.09 (8)
N1—C7—H7	121.1	C11—N2—H2A	120.0
C6—C7—H7	121.1	C11—N2—H2B	120.0
O2—C9—O1	122.17 (9)	H2A—N2—H2B	120.0
O2—C9—C10	125.21 (9)	C9—O1—N1	110.62 (7)
O1—C9—C10	112.61 (8)		
			
C6—C1—C2—C3	0.25 (16)	C15—C10—C11—N2	−178.31 (10)
Cl1—C1—C2—C3	179.50 (8)	C9—C10—C11—N2	2.12 (15)
C1—C2—C3—C4	−0.16 (16)	C15—C10—C11—C12	0.59 (14)
C2—C3—C4—C5	0.48 (16)	C9—C10—C11—C12	−178.99 (9)
C3—C4—C5—C6	−0.89 (16)	N2—C11—C12—C13	178.24 (10)
C2—C1—C6—C5	−0.63 (15)	C10—C11—C12—C13	−0.71 (15)
Cl1—C1—C6—C5	−179.86 (8)	C11—C12—C13—C14	0.47 (16)
C2—C1—C6—C7	179.90 (9)	C11—C12—C13—Cl2	−179.67 (8)
Cl1—C1—C6—C7	0.66 (14)	C12—C13—C14—C15	−0.08 (16)
C4—C5—C6—C1	0.95 (15)	Cl2—C13—C14—C15	−179.94 (8)
C4—C5—C6—C7	−179.57 (9)	C13—C14—C15—C10	−0.04 (15)
C1—C6—C7—N1	−160.77 (10)	C11—C10—C15—C14	−0.22 (15)
C5—C6—C7—N1	19.77 (14)	C9—C10—C15—C14	179.35 (9)
O2—C9—C10—C15	175.68 (10)	C6—C7—N1—O1	−178.86 (8)
O1—C9—C10—C15	−5.26 (14)	O2—C9—O1—N1	−4.58 (14)
O2—C9—C10—C11	−4.74 (16)	C10—C9—O1—N1	176.34 (8)
O1—C9—C10—C11	174.31 (9)	C7—N1—O1—C9	168.81 (9)

### Hydrogen-bond geometry (Å, º)

**Table:**

*D*—H···*A*	*D*—H	H···*A*	*D*···*A*	*D*—H···*A*
N2—H2*B*···O2	0.88	2.02	2.6653 (12)	130
N2—H2*A*···O2^i^	0.88	2.19	2.9332 (12)	142

Symmetry code: (i) *x*+1/2, −*y*+1/2, *z*+1/2.
